# Epstein–Barr Virus and Innate Immunity: Friends or Foes?

**DOI:** 10.3390/microorganisms7060183

**Published:** 2019-06-24

**Authors:** Sonia Jangra, Kit-San Yuen, Michael George Botelho, Dong-Yan Jin

**Affiliations:** 1 Department of Prosthodontics, Faculty of Dentistry, The University of Hong Kong, Sai Ying Pun, Hong Kong, China; sonia.jangra368@gmail.com (S.J.); botelho@hku.hk (M.G.B.); 2 School of Biomedical Sciences, Li Ka Shing Faculty of Medicine, The University of Hong Kong, Pokfulam, Hong Kong, China; yuenkitsan0119@gmail.com

**Keywords:** Epstein–Barr virus, EBV, interferon, inflammasome, caspase

## Abstract

Epstein–Barr virus (EBV) successfully persists in the vast majority of adults but causes lymphoid and epithelial malignancies in a small fraction of latently infected individuals. Innate immunity is the first-line antiviral defense, which EBV has to evade in favor of its own replication and infection. EBV uses multiple strategies to perturb innate immune signaling pathways activated by Toll-like, RIG-I-like, NOD-like, and AIM2-like receptors as well as cyclic GMP-AMP synthase. EBV also counteracts interferon production and signaling, including TBK1-IRF3 and JAK-STAT pathways. However, activation of innate immunity also triggers pro-inflammatory response and proteolytic cleavage of caspases, both of which exhibit proviral activity under some circumstances. Pathogenic inflammation also contributes to EBV oncogenesis. EBV activates NFκB signaling and induces pro-inflammatory cytokines. Through differential modulation of the proviral and antiviral roles of caspases and other host factors at different stages of infection, EBV usurps cellular programs for death and inflammation to its own benefits. The outcome of EBV infection is governed by a delicate interplay between innate immunity and EBV. A better understanding of this interplay will instruct prevention and intervention of EBV-associated cancers.

## 1. Introduction

Epstein–Barr Virus (EBV), also known as human herpesvirus 4 (HHV-4), is a member of the subfamily of *Gammaherpesvirinae*, which also includes Kaposi sarcoma-associated herpesvirus (KSHV). EBV infects more than 95% of adults worldwide. EBV is transmitted through saliva and primarily infects B cells and epithelial cells, but macrophages and dendritic cells also play important roles in EBV infection. EBV is associated not only with oral diseases such as infectious mononucleosis and oral hairy leucoplakia but also with several types of epithelial cell carcinoma, such as nasopharyngeal carcinoma (NPC) and gastric carcinoma, and with B cell lymphoma, including Burkitt lymphoma, posttransplant lymphoproliferative disorder, and Hodgkin and non-Hodgkin lymphoma [1]. EBV establishes latency in the host cells after primary infection, which is a typical characteristic of a gammaherpesvirus. The viral genetic material replicates along with the host genome. Lytic reactivation can be induced by the expression of viral BZLF1 protein, also known as Zta, leading to virion production and the spread of EBV infection. Both lytic and latent phases are required in the life cycle of EBV. Whereas EBV-associated malignancies develop only in latently infected cells, lytic replication is thought to be required for EBV oncogenesis [2]. The lytic-latent switch is an important event in EBV infection, but its regulatory mechanism remains to be fully understood [3,4,5].

At least three different latency states of EBV have been defined based on different expression patterns of latent genes. During latency III, B cells are transformed into immortalized lymphoblastoid cell lines expressing six EBV nuclear antigens (EBNAs), three latent member proteins (LMPs), and several noncoding RNAs (ncRNAs), including EBV-encoded RNAs (EBERs), BamHI A rightward transcripts (BARTs), and EBV-encoded microRNAs (miRNAs). Latency II occurs in NPC cells, and the expression of EBV genes is restricted to EBNA1, LMPs, and ncRNAs. In contrast, typical Burkitt lymphoma cells are in latency I, where only EBNA1 and ncRNAs are expressed [6,7]. In addition, another special latency program known as Wp-restricted latency can be established by EBNA2-deleted EBV in Burkitt lymphoma cells [8,9]. In this state, EBNA1, EBNA3s, and EBNA-LP are expressed from a Wp promoter rather than a Qp promoter. BCL2 homolog BHRF1 is also expressed.

During viral infection, viral constituents containing pathogen-associated molecular patterns (PAMPs) are recognized by pattern recognition receptors (PRRs) of the infected cell, hence stimulating innate antiviral immune response. This response results in the production and release of various cytokines including interleukins (ILs), tumor necrosis factor (TNF), and interferons (IFNs) from the infected cells. Type I IFN response is one of the vital antiviral defense mechanisms of the host cells. The major PRRs consist of membrane-bound and cytoplasmic sensors, which can be subdivided into several protein families including Toll-like receptors (TLRs), RIG-I-like receptors (RLRs), NOD-like receptors (NLRs), and AIM2-like receptors (ALRs). In addition, cyclic GMP-AMP (cGAMP) synthase (cGAS) is another key sensor of cytoplasmic DNA. Activation of PRRs by PAMPs triggers not only JAK-STAT-mediated IFN response but also different branches of innate immune signaling including NFκB pathway; inflammasome activation; and programmed cell death such as apoptosis, necroptosis, and pyroptosis [10,11].

To evade innate immune sensing and the consequent activation of antiviral cascades, EBV has evolved multiple effective countermeasures. These can occur at different pathways and steps ranging from recognition by cell surface, endosomal, and intracellular sensors to IFN production and signaling. This interplay between EBV and innate immunity is influential to the outcome of infection. The main theme is to promote viral replication and to sustain viral infection. However, innate immunity is a double-edge sword as the induction of pro-inflammatory responses and activation of programmed cell death might release a burst of virions and may therefore facilitate the spread of infection [12]. Furthermore, activation of caspases might serve a proviral role in the lytic replication of EBV through proteolytic cleavage of critical cellular and viral proteins [13]. Further investigations are required to elucidate how inflammasome activation, programmed cell death, and caspase activation fulfill their proviral function in the context of EBV infection. In this review, we will discuss the current knowledge of countermeasures adopted by EBV to perturb innate immune response with a focus on IFN production and signaling, inflammasome assembly, programmed cell death, and caspase activation.

## 2. EBV Perturbation of PRRs

### 2.1. TLR Signaling

Most viruses, including herpesviruses, stimulate innate immune response during primary infection predominantly by activating TLRs. TLRs activate downstream adaptor molecules such as MyD88, TIRAP, TRAFs, TRIF, and TRAM to induce type I IFNs [14,15,16,17]. These adaptor molecules form multiprotein complexes containing TBK1 kinase to induce the activation of IRF3 and IRF7 transcriptional factors, which induce type I IFN production. NFκB is another downstream effector of TLR signaling. EBV is capable of modulating various TLRs and TRAFs to perturb IFN production and NFκB activation.

**Figure 1:**
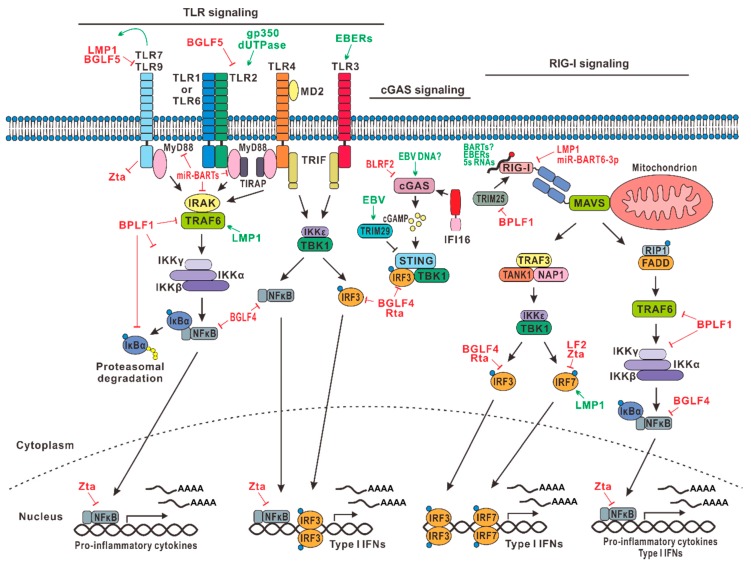
Epstein–Barr virus (EBV) perturbation of Toll-like receptor (TLR), RIG-I-like receptor (RLR), and cyclic GMP-AMP (cGAMP) synthase (cGAS) signaling. Signaling through TLR (left part of the diagram), RIG-I (right part), and cGAS (central part) is positively and negatively regulated by EBV proteins and RNAs. On the one hand, EBV proteins and RNAs inhibit these pathways to suppress antiviral interferons (IFN) and cytokine production to facilitate viral infection (highlighted in red). On the other hand, they can also activate some of these pathways to promote cellular growth and survival (shown in green). See text for further details.

Viral constituents can be recognized by TLRs expressed at the cell surface, in the endosomal membrane, or in the cytoplasm. As such, TLR2, TLR3, TLR4, TLR7, TLR8, and TLR9 are known to recognize EBV during infection [18,19]. The expression and activity of TLRs in infected cells are activated by EBV [20]. In addition, during the lytic cycle, EBV also activates TLR signaling in pDCs. TLR2 detects herpesviruses such as herpes simplex virus 1, cytomegalovirus, varicella-zoster virus, and EBV [21,22]. During primary infection, TLR2 is likely activated by EBV surface glycoprotein gp350 (Figure 1), which also binds to cellular receptor CD21 to mediate viral entry and membrane fusion [23]. Table 1 provides a summary of the EBV genes discussed in this review. Another PAMP of TLR2 is the EBV-encoded dUTPase, which is a nonstructural protein and an early antigen [24]. EBERs produced in the infected cells are detected by TLR3 [25]. EBV can activate monocytes and plasmacytoid dendritic cells (pDCs) through cooperative action of TLR9 and TLR2 [26]. TLR9 is the major TLR present in B cells and is responsible for the production of IFNs, IL-6, TNF-α, and immunoglobulins from infected cells. TLR9 can also recognize CpG motifs in dsDNA and, therefore, can detect DNA genome of EBV and murine gammaherpesvirus 68 (MHV68), which serves as a model for both EBV and KSHV [27]. Endosomal TLR7 and TLR9 in pDCs also cooperate with each other to sense MHV68 infection [28]. In this regard, the ligand of TLR7 is single-stranded RNA. Interestingly, TLR9 is known to inhibit EBV lytic replication by suppressing BZLF1 [29].

EBV is capable of regulating TLRs differentially, depending on the phase of life cycle and the stage of viral egress. Whereas robust amplification and replication of EBV genome during the lytic phase require more efficient suppression of host antiviral response, EBV also has to evade innate recognition and clearance persistently during latency. During primary infection, EBV downregulates TLR7, TLR8, and TLR9 expression to support viral replication in infected B cells [30]. Multiple lytic proteins are employed to antagonize TLR signaling. TLR9 mRNA is targeted for degradation in EBV-infected B cells by EBV lytic protein BGLF5, leading to reduced production of TLR9 [31]. BGLF5, being a viral alkaline exonuclease, also targets TLR2 by reducing its expression in the infected cells [32]. Additionally, another EBV-encoded protein BPLF1, a deubiquitinase and a late lytic tegument protein, interferes with the ubiquitination and activation of signaling intermediates in the TLR pathway, thereby inhibiting TLR-mediated IFN production in infected cells [33]. During latency, EBV also suppresses TLR2-, TLR5-, and TLR9-mediated IFN production. In particular, EBV-encoded major oncogenic protein LMP1 expressed in latency II and III cells targets TLR9 by inhibiting its promoter activity [34]. In addition, EBV-encoded miRNAs including miR-BARTs are also thought to be capable of suppressing TLR signaling [35].

TRAFs are adaptor proteins in TLR signaling. TRAF6 is important in the maintenance of EBV latency and the inhibition of lytic cycle progression. The impact of EBV-encoded LMP1 on TLR-TRAF signaling has been well-characterized. LMP1 has two cytoplasmic domains called CTAR1 and CTAR2, which have binding sites for TRAFs. LMP1 recruits and activates TRAF2, TRAF3, and TRAF6, thereby activating downstream NFκB signaling to promote cell growth and survival. EBV-encoded lytic protein BPLF1 inhibits TRAF6 activation through its deubiquitinase activity (Figure 1), leading to the suppression of IFN production and NFκB activation [33,35,36]. This favors lytic replication or reactivation. In other words, inhibition of TRAF6 serves to trigger the latent-lytic switch.

EBV perturbation of TLR signaling during lytic and latent phases is dynamic and under stringent control. TLR signaling is activated transiently after viral entry but has to be effectively suppressed subsequently. Enforced activation of TLR signaling using a TLR9 agonist prevents initiation of the EBV lytic cycle [37]. Interestingly, TLR7 has been shown to activate LMP1 [38], which in turn suppresses TLR9 as described above [34]. Thus, they possibly constitute a negative feedback loop. TLR9 polymorphism is influential in the susceptibility of immunocompromised infants to EBV infection [39]. This lends support to the notion that TLR signaling is critical in EBV infection. Whereas the antiviral effect of TLR signaling is effectively suppressed by EBV, the pro-inflammatory response triggered by TLR signaling is still prominent under some circumstances. Chronic inflammation as a result of EBV infection not only contributes to EBV pathogenesis and oncogenesis but also exhibits a proviral effect. Thus, EBV and innate immunity have reached a mutualistic equilibrium after a long history of coevolution [40,41].

### 2.2. RLR and cGAS Signaling

Although viruses are detected by TLRs during their entry into target cells, the nucleic acid sensors in the cells detect viral DNA and RNA that have already successfully entered the cells. As a DNA virus, EBV is thought to be recognized primarily by DNA sensors. The viral DNA is detected by cytoplasmic DNA sensors. Although different sensors including IFI16, DDX41, and DAI have been suggested to play a role in the detection of viral DNA, cyclic GMP-AMP (cGAMP) synthase (cGAS) is now well-recognized as the master DNA sensor, which produces a cGAMP second messenger to bind and activate STING, leading ultimately to type I IFN production [42,43]. Interestingly, other sensors such as IFI16 either cooperate with or operate through cGAS [44,45,46]. In addition, EBV also encodes RNAs that can be detected by cytoplasmic RNA sensors such as RIG-I [47]. All these PRRs activate downstream TBK1 signaling, which is largely overlapping with that trigged by activation of TLRs [48,49].

#### 2.2.1. RLR Signaling

RLRs are intracellular PRRs that detect cytoplasmic RNAs. RLRs include RIG-I, MDA5, and LPG2, among which RIG-I is known to play a role in sensing EBV infection. Particularly, EBERs are recognized by RIG-I [47]. EBERs including EBER1 and EBER2 are abundantly expressed in EBV-infected cells. As such, laboratory detection of EBERs serves as a defining diagnostic test for EBV infection. EBERs are highly structured, and it is therefore not surprising that EBERs activate RIG-I signaling. ERERs can activate other RNA sensors such as TLR3 described above [25] and can also suppress PKR [50,51]. Whether the BART transcripts abundantly expressed in EBV-infected cells and particularly epithelial cells might also trigger RIG-I signaling remains to be determined. In addition, two lines of loss-of-function experiments would help to fully characterize the activation of RIG-I by EBERs. First, a recombinant EBV that either does not produce EBERs or produces significantly less amounts of EBERs should show a difference in its ability to activate RIG-I in a physiologically relevant cell line. Second, the induction of type I IFN by EBV should be examined in RIG-I^−/−^ cells. The function of EBERs remains incompletely understood, and it will be of great interest to see whether their major role in infected cells is to serve as a PAMP. One report suggests that EBER1 binds with cellular La ribonucleoprotein in B cells to evade sensing by RIG-I. In addition, EBER1 carrying 5′-triphosphates in infected B cells can be transferred via exosomes to nonpermissive pDCs to drive innate immune sensing [52]. One interesting alternative mechanism through which EBV activates innate immunity is to unmask cellular 5S rRNA pseudogene transcripts for recognition by RIG-I [53]. This unmasking also occurs during infection with HSV-1 and influenza A virus.

EBV can inhibit RIG-I-mediated sensing of viral RNAs in infected cells to prevent the induction of type I IFNs. EBV-encoded LMP1 promotes RIG-I degradation via the proteasome-degradation pathway by recruiting an E3 ubiquitin ligase named CHIP to RIG-I [54]. EBV-encoded deubiquitinase BPLF1 can bind to another E3 ligase TRIM25, which ubiquitinates RIG-I at K63, resulting in its binding with MAVS and in the activation of downstream signaling. BPLF1 forms a ternary complex with TRIM25 and 14–3–3 scaffold protein to promote auto-ubiquitination of TRIM25, which reduces ubiquitination of RIG-I and dampens RIG-I signaling [55]. Furthermore, EBV also encodes miRNAs to inhibit the RIG-I pathway. EBV-encoded miBART6-3p targets RIG-I mRNA, resulting in the inhibition of type I IFN expression and the consequent reduction of phospho-STAT1 levels in peripheral blood mononuclear cells [56]. Additional RIG-I-targeting miR-BARTs might also be identified and characterized. It is seemingly contradictory that the primary transcripts of EBV-encoded RNA such as EBERs activate RIG-I, whereas the mature viral miRNAs exert an inhibitory effect (Figure 1). Further investigations are required to clarify the net effect of EBV-encoded noncoding RNAs on RIG-I signaling and, more importantly, the relevance to EBV biology.

#### 2.2.2. cGAS-STING

Although the cGAS-STING signaling pathway of innate DNA sensing is more relevant to EBV, studies on EBV DNA sensing or EBV perturbation of DNA sensing are scarce and should be strengthened. Currently, it is assumed that EBV will be sensed in the same way as any other herpesvirus or gammaherpesvirus. EBV-specific mechanisms of sensing and anti-sensing remain to be elucidated. 

Herpesviruses can activate cGAS-STING signaling [42,57,58,59]. For example, MHV68 can activate cGAS-mediated DNA sensing. For the benefit of their own replication, gammaherpesviruses have to develop strategies to bypass or suppress cGAS signaling. An abundantly expressed KSHV tegument protein ORF52 as well as its homologs in EBV and MHV68 are known to interact with cGAS, hindering its capacity to bind with viral dsDNA [60,61]. EBV has also been shown to induce cellular E3 ligase TRIM29 to induce K48-linked ubiquitination and degradation of STING (Figure 1), preventing the activation of cGAS-STING signaling [62,63]. One recent study suggests that human B cells are dysfunctional in cGAS-STING signaling. As such, EBV-transformed B cells cannot produce type I IFNs upon stimulation with dsDNA or cGAMP [64]. It remains to be fully understood how this defect might be relevant to EBV infection of B cells. In this regard, several important questions need to be addressed experimentally:

- Is cGAMP production activated by EBV in infected B cells and epithelial cells?
- Is EBV capable of suppressing cGAS-STING signaling in a physiologically relevant context?
- Is there a real difference between EBV-infected B cells and epithelial cells in the sensing of EBV DNA?
- What is end result of the interplay between EBV and DNA sensing?

### 2.3. NLR and ALR Signaling

NLRs are PRRs that contain a nucleotide-binding oligomerization domain (NOD). NLRs can recognize a wide range of PAMPs and danger signals. ALRs are PRRs that are structurally related to AIM2 containing an N-terminal pyrin domain (PYD) and a C-terminal HIN domain [65]. Whereas PYD helps to recruit the inflammasome adapter protein ASC through PYD–PYD interaction, the positively charged HIN is a dsDNA-binding domain.

**Figure 2:**
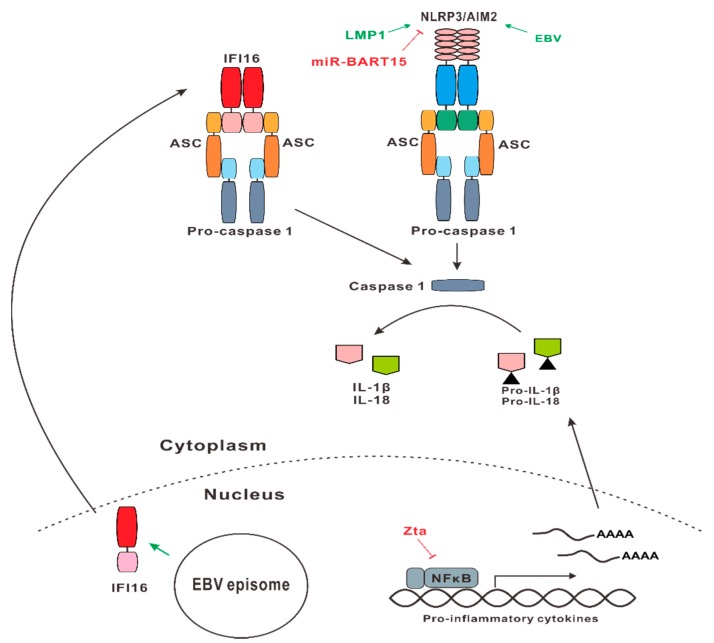
EBV perturbation of NOD-like receptor (NLR) and AIM2-like receptors (ALR) signaling. On the one hand, EBV suppresses these pathways through its viral proteins and RNAs to circumvent their antiviral effects (shown in red). On the other hand, EBV can also activate some of these pathways to cause pathogenic inflammation and to facilitate viral spreading and infection (highlighted in green). See text for further details.

One NLR and two ALRs have currently been implicated in host–EBV interaction (Figure 2). While NLRP3 is the NLR that has been shown to be modulated by EBV oncoprotein LMP1 [66], AIM2 and IFI16 are the two ALRs that are known to be dysregulated by EBV [67,68]. Activation of NLRP3, AIM2, and IFI16 by EBV PAMPs such as its DNA genome results in the assembly of inflammasome. Inflammasome is the multiprotein complex formed to recruit the essential adaptor protein ASC, which recruits procaspase 1 through CARD–CARD interaction, leading to proteolytic activation of pro-inflammatory cytokines IL-1β and IL-18 [69].

Inflammasome activation might serve both antiviral and proviral roles during EBV infection. As part of the innate antiviral response, it could help to restrict EBV replication and infection. However, it might also promote viral dissemination by releasing a large amount of infectious EBV virions. In addition, when myeloid cells have migrated to the inflammation site, EBV will spread to dendritic cells and macrophages; this serves an important function in EBV infection in vivo [69].

There is only circumstantial evidence in support of the activation of NLRP3 inflammasome by LMP1 in association with the expansion of myeloid-derived suppressor cells in the tumor microenvironment of NPC [66]. In another perspective, NLRP3 activation is induced by extracellular ATP and reactive oxygen species in EBV-associated NPC, whereas AIM2 is required for IL-1β production induced by EBV DNA in infected epithelial cells. As a result of IL-1β secretion, neutrophil recruitment is enhanced, which serves as a favorable prognostic marker for local recurrence-free survival [70]. The activation of AIM2 but not NLRP3 or IFI16 is also seen in EBV-infected THP-1 cells, which results in IL-1β secretion [67]. On the other hand, EBV-encoded miR-BART15 has been shown to inhibit NLRP3 and IL-1β maturation (Figure 2). Interestingly, miR-BART15 can be secreted by infected B cells via exosomes to inhibit NLRP3 activation in noninfected cells [71]. In addition, EBV-encoded miR-BHRF1-2-5p targets IL-1 receptor 1 to prevent the action of IL-1α and IL-1β [72]. It will be of interest to see at what stage of infection and through what mechanism EBV mobilizes its activators and inhibitors of inflammasome activation to avoid dangers and to gain benefits.

The genome of HSV-1 is also thought to be detected by IFI16 in infected cells. Whereas AIM2 is a cytoplasmic protein, IFI16 localizes primarily to the nucleus. As such, IFI16 co-localizes with EBV and KSHV genome in infected cells. IFI16 contains two C-terminal HIN domains in addition to the N-terminal pyrin domain. The HIN domains named HIN A and HIN B bind dsDNA cooperatively [73]. This led to the hypothesis that IFI16 might act as a nuclear sensor of EBV genome with the ability to distinguish nonself from self-DNA [68,74]. This results in the constitutive activation of an IFI16-ASC-caspase-1 inflammasome and AIM2-independent production of IL-1β [74,75,76,77]. Activation of IFI16 could also lead to pyroptosis of infected cells [78]. Notably, the distribution of IFI16 in the cytoplasm of macrophages and lymphocytes could enable it to sense DNA, to facilitate cGAMP synthesis by cGAS, and to activate STING-dependent IFN production [45]. IFI16 shuttles dynamically between the nucleus and the cytoplasm under the control of acetylation of its nuclear localization signal (Figure 2). In this sense, IFI16 functions as a DNA sensor in both the nucleus and the cytoplasm [79].

However, although IFI16 might be a restriction factor for EBV and other herpesviruses, its exact role in EBV infection and the dependence on IFN signaling remain controversial. In this regard, three lines of evidence are noteworthy. First, as mentioned above [44,45,46], IFI16 interacts with cGAS and might rely upon cGAS-STING signaling to exert its influence on IFN production [80,81]. Second, IFI16 accumulates on the genome of herpesviruses and modulates viral gene expression through histone modifications [82]. Finally, IFN response to cytoplasmic DNA and DNA virus remains intact in mice deficient in p204, which is the mouse counterpart of IFI16, and all other 12 ALRs. In other words, p204 is nonessential for DNA-induced type I IFN production or sensing of DNA viruses in vivo [83]. Thus, further investigations are required to elucidate exactly how IFI16 is involved in the EBV life cycle.

### 2.4. TLR and RLR Effectors

TLR and RLR signaling converges to activate NFκB and IRF3/IRF7 transcription factors. Upstream of this, transducer proteins including MAVS and TBK1 are also shared by different pathways. All these steps are also perturbed by EBV. Ensuring an effective and accurate suppression of innate antiviral response would be one reason for EBV to target these common effectors.

#### 2.4.1. NFκB Pathway

NFκB is a master transcription factor in immunity and inflammation. It is highly influential in EBV life cycle and oncogenesis. NFκB inhibits the lytic replication of EBV [84] through repression of the expression of lytic inducers Zta and Rta [85,86]. One mechanism through which EBV activates NFκB is through the recognition of EBV virions or surface glycoprotein gp350 by host TLRs via a MyD88-dependent signaling pathway. This results in the induction of cytokine expression in infected cells [87].

To establish and maintain latency in infected cells, EBV oncoprotein LMP1 activates NFκB pathway through its cytoplasmic domains CTAR1 and CTAR2. LMP1 is a member of the TNF receptor superfamily, and it mimics CD40 to activate NFκB and other growth and survival pathways [88,89]. The CTAR1 domain of LMP1 directly recruits TRAF1, TRAF2, TRAF3, and TRAF5 to activate the IKKα-dependent NFκB pathway, whereas the CTAR2 domain of LMP1 indirectly recruits TRAF6 to activate the IKKβ- and IKKα-dependent NFκB pathways [90,91]. NFκB signaling promotes growth and survival of the infected cells [36,92,93]. Through NFκB activation, LMP1 also induces IL-1α, IL-1β, and TNF-α in infected cells, favoring transformation as in the context of NPC development [94,95].

On the other hand, the NFκB pathway is downregulated during lytic replication of the virus. EBV deubiquitinase BPLF1 exerts an inhibitory effect on TRAF6 to prevent NFκB activation [33,36,47]. Early lytic transactivator Zta induces p65 nuclear translocation but inhibits its transcriptional activity [96]. It also binds to TNF-α promoter to impede the function of NFκB [97]. Rta of MHV68 promotes ubiquitination and degradation of RelA, a subunit of NFκB. It also inhibits MAVS-dependent NFκB activation [98,99]. BGLF4 kinase of EBV can also suppress NFκB by targeting an essential coactivator UXT [86]. In addition, EBV-encoded miR-BART6-3p and miR-BHRF1-2-5p downregulate NFκB by targeting IL-6 receptor and IL-1 receptor, respectively during latency or lytic reactivation [72,100]. Several EBV-encoded miRNAs are also known to perturb B cell receptor engagement, thereby attenuating downstream NFκB signaling [101]. Generally, EBV promotes NFκB activation during latency to maintain cell growth but inhibits NFκB signaling during the lytic replication cycle.

#### 2.4.2. IRF3 and IRF7

IRF3 and IRF7 are the principal transcription factors that activate IFN production in response to the activation of TLRs, RLRs, and other PRRs [102]. IRF3 and IRF7 are also important during EBV infection and transformation. Interestingly, IRF7 was initially discovered as a negative regulator of the Qp promoter of EBV, from which EBNA-1 mRNA is transcribed during latency I [103]. IRF7 is also known to be induced by EBV oncoprotein LMP1 [104]. Apparently, IRF3 and IRF7 might not just be inhibited during EBV infection, and at some stages, they could also be activated and can serve proviral or pro-transformation roles. It will be of great interest to see whether their induction might be influential in different latency states of EBV and in EBV oncogenesis. On the other hand, their inhibition during lytic replication is required for proper progression of the lytic cycle.

Indeed, during lytic replication, IRF3 is targeted by EBV-encoded BGLF4 and Rta. BGLF4 is a protein kinase, which directly interacts with and phosphorylates IRF3 to impede formation of a stable IRF3–DNA complex. Therefore, BGLF4 inhibits type I IFN expression, resembling its herpes simplex virus 1 ortholog UL13 [105]. Rta also downregulates IRFs in the infected cells. EBV-encoded Rta has been suggested to suppress IRF3 expression in HeLa cells by inducing the E2F1 transcription factor that binds to the IRF3 promoter [106]. However, exactly how Rta induces E2F1 and how E2F1 represses IRF3 expression in a physiologically relevant context remain elusive. It will be of interest to see whether Rta reduces the production or promotes the degradation of IRF3 and IRF7. In this regard, KSHV-encoded Rta exhibits E3 ubiquitin ligase activity that targets IRF7 and other key transducers in innate immune signaling such as MyD88 and TRIF, which are critical in TLR signaling, for degradation [107,108,109]. Therefore, Rta proteins of gammaherpesviruses negatively regulate type I IFN responses to facilitate viral replication [110]. Whether EBV Rta might suppress IRF7 and innate immune signaling through an intrinsic E3 activity like its distantly related counterpart in KSHV merits further investigations. In addition, IFN antagonism of another key lytic inducer Zta and tegument protein LF2 of EBV has also been documented. Both directly interact with IRF7 to inhibit its dimerization but not nuclear translocation or phosphorylation [111,112].

As mentioned above, the expression pattern of IRF7 in latency is opposite to that during lytic replication. Induction of IRF7 and type I IFN by LMP1 is thought to prime cells for the establishment of latency [113,114]. Furthermore, LMP1 activates IRF7 by enhancing K63-linked ubiquitination of IRF7 by RIP [115]. To counterbalance the effect of IRF7, LMP1 also induces the expression of an IRF7C splice isoform that has an inhibitory effect, adding another level of complexity [116].

The diagrams in Figure 1 and Figure 2 depict the perturbations of TLR, NLR, RLR, ALR, and cGAS signaling by EBV.

## 3. EBV Perturbation of IFN Signaling

**Figure 3:**
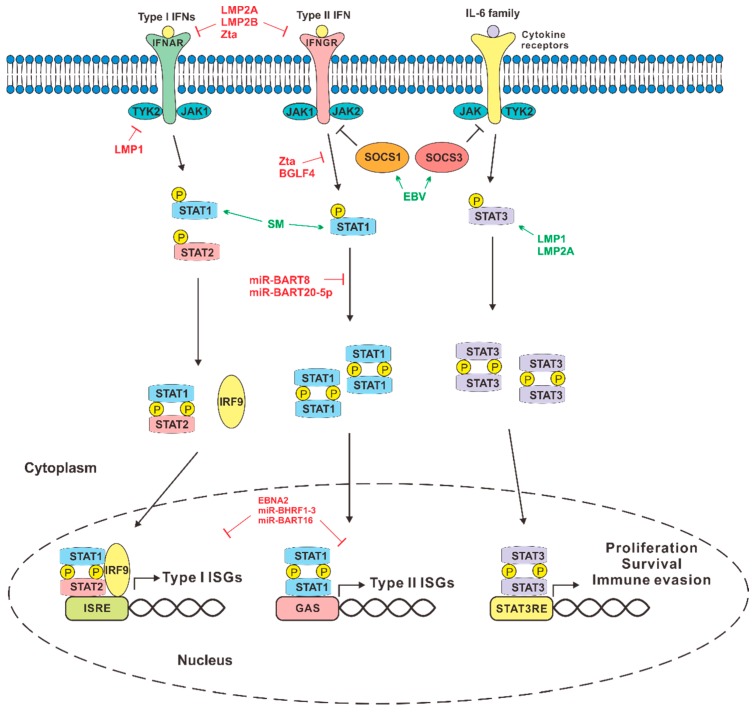
EBV perturbation of IFN signaling. EBV proteins and RNAs suppress Type I and Type II IFN signaling to facilitate EBV infection, maintenance, and reactivation in the infected cells (molecules depicted in red). Some of them can also activate STAT signaling, including STAT3, to promote cell proliferation, survival, and immune evasion (molecules depicted in green). STAT3RE: STAT3 response element. See text for further details.

When expressed in cells, IFNs execute their antiviral functions through JAK-STAT signaling. It is therefore not surprising that EBV has developed multiple strategies to counteract not only IFN production but also IFN signaling. JAK-STAT pathways are activated when IFNs produced by virus-infected cells bind to their specific receptors. Type I IFNs including IFN-α, -β, -ε and other subtypes interact with IFNAR1 and IFNAR2, which are respectively associated with TYK2 and JAK1 tyrosine kinases, which phosphorylate and activate downstream transcription factors STAT1 and STAT2 (Figure 3). Together with IRF9, a ternary complex of STAT1-STAT2-IRF9, also called ISGF3, is formed to stimulate the transcription of interferon-stimulated genes (ISGs) such as MX-A, ISG15, ISG56, and OAS1 through IFN-stimulated response elements (ISREs) [117]. Type III IFNs such as IFN-λ bind to receptors IL-10-R2 and IFNLR1 to activate JAK1 and TYK2. On the other hand, IFN-γ is recognized by IFNGR1 and IFNGR2, leading to the activation of JAK1 and JAK2. These kinases phosphorylate and activate STAT1 to form homodimers, also known as GAF [118]. This dimer translocates into the nucleus to induce the transcription of ISGs such as IP10, IRF1, EGR1, CIITA, and CCL2S through enhance elements named gamma activated sequence (GAS) [117].

It is not surprising that type I IFNs can inhibit the replication of gammaherpesviruses in infected cells [119]. Perturbation of IFN signaling by EBV is generally seen as a protective and adaptive mechanism to ensure successful infection and other benefits to the virus.

### 3.1. JAK-STAT Signaling

JAK-STAT signaling is triggered by the binding of IFNs to their receptors. EBV proteins target IFN receptors to prevent IFNs from binding and activating their receptors. Particularly, LMP-2A/2B and Zta increase the turnover of IFNARs and IFNGRs, thus inhibiting IFN response [120,121].

STATs are the transcription factors that are phosphorylated and activated by upstream JAK kinases. JAKs and STATs are common targets of EBV proteins and miRNAs. We discussed above that LMP1 has a negative impact on TLR signaling [34]. It also induces IRF7 to activate IFN response [113,114]. In addition, LMP1 has an N-terminal transmembrane domain, which directly interacts with TYK2 kinase involved in type I and type III IFN signaling and suppresses phosphorylation of both STAT1 and STAT2 (Figure 3). Therefore, LMP1 can block IFN-mediated antiviral response in infected cells [122]. These findings are seemingly at odds with each other. Whether the different observations might be explained by the cell type or experimental setting remains to be determined. It will also be of great interest to see whether LMP1 might differentially regulate IFN production and signaling, leading to the induction of specific subsets of ISGs. Nevertheless, LMP1 might have dual regulatory roles and may selectively activate or repress some ISGs to facilitate viral infection.

EBV also targets type II IFN signaling by altering STAT1 levels and preventing transcription of ISGs. Lytic trans-activator Zta inhibits tyrosine phosphorylation of JAK1 and JAK2. Zta and protein kinase BGLF4 of EBV inhibit Tyr701 phosphorylation of STAT1, inhibiting its nuclear translocation [105,121]. Therefore, there is no induction of ISGs in response to IFN-γ. In addition, EBV miRNAs can also inhibit JAK/STAT signaling. miR-BART 20-5p and miR-BART8 are known to suppress type II IFN signaling, whereas miRNA-BART8 also inhibits STAT1 translation in cells, therefore affecting both type I and type II IFN signaling [123,124].

On the contrary, SM, also called BMLF1/EB2/MTA, which is an early lytic nuclear protein and a posttranscriptional gene activator, induces the expression of STAT1, particularly STAT1-β splice variant, and thus increases the expression of ISGs. Although SM does not directly affect IFN-α/β production [125], it might induce STAT1 possibly as a transcription factor to facilitate the transcription of EBV genes. Several unanswered questions concerning the roles and mechanism of SM in EBV replication and innate immune modulation arise when we consider this finding together with current literature. SM can inhibit several viral and cellular regulators of viral replication and innate immunity including RNA helicase DHX9 [126], which is known to be important in the repression of innate immunity. In particular, DHX9 is critically involved in DNA and RNA sensing [127,128]. It also interacts with MAVS and activates NFκB and TLR signaling [129,130]. Whether SM might promote DHX9 function remains to be elucidated. On the other hand, SM has a more general stimulatory effect on translational initiation [131]. Whether its induction of STAT1 is specific requires further investigations. Assuming that SM does induce STAT1 and ISGs, the relevance of this induction to EBV infection remains to be clarified. Whether this occurs at an early stage of primary infection and mediates the initial burst of IFNs and cytokines awaits further analysis. In this regard, transcriptomic analysis would be very helpful in determining the subset of ISGs induced by SM.

In addition to STAT1 and STAT2, other STATs also play a role in cytokine signaling. They could also form heterodimers with STAT1, which might possess unique transcriptional activity. These other STATs have also been shown to be activated by EBV^+^ leukemic cells [132]. Particularly, STAT3 is selectively activated by LMP1 [133,134,135]. In addition, LMP2A can also induce IL-10 through activation of STAT3 [136]. One functional outcome of STAT3 activation by EBV might be autophagy of infected cells [135]. Nevertheless, the restriction of EBV latency as a result of B cell-specific knockout of STAT3 not only provides in vivo support to the importance of STAT3 in EBV latency establishment but also suggests that targeting STAT3 might have beneficial effects in EBV-associated B cell malignancies [137].

### 3.2. ISGs

EBV proteins capable of inhibiting IFN signaling such as Zta, BGLF4, and LMP1 necessarily affect the expression of ISGs, thereby preventing the establishment of an antiviral state and promoting viral replication. However, the orthodoxical view of all ISGs being antiviral has been challenged. Indeed, some ISGs are proviral or have dual activities [138]. Thus, EBV proteins might differentially modulate the expression of ISGs. Some of them are also capable of differentially regulating IFN production and signaling. For example, although EBNA2 can induce a low amount of type I IFNs, it still prevents the induction of ISGs. Hence, EBNA2 might induce inflammation mediated by IFNs but might prevent the establishment of the antiviral state through inhibition of ISG expression [139,140].

Several EBV miRNAs also contribute to the suppression of ISG expression. EBV-encoded miRNA BHRF1-3 suppresses the expression of CXCL11, an ISG important for T cell activation [141]. In addition, miR-BART20-5p and miR-BART8 inhibit IFN-γ signaling by reducing the levels of phosphor-STAT1 [94]. miR-BART16 inhibits IFN and ISG expression by targeting CREB binding protein (CBP), a transcriptional coactivator [142]. Since the effect of CBP on transcription should not be specific to IFNs and ISGs, it remains to be seen how targeting CBP could exert a specific suppressive effect on IFN response.

### 3.3. SOCS Proteins

IFNs induce suppressor of cytokine signaling (SOCS) proteins as a negative feedback mechanism to regulate IFN signaling. As such, type I IFNs upregulate SOCS1, SOCS3, and USP18 to tune down IFN signaling and ISG induction. EBV can activate SOCS3 in infected cells to inhibit type I IFN secretion, providing an additional mechanism to keep IFN signaling in check [143]. Apart from encoding viral miRNAs, EBV also upregulates cellular miRNAs. EBV-induced cellular miRNA155 downregulates SOCS1 and induces STAT3, which induces proliferation and inflammation in favor of cellular transformation (Figure 3). It also induces cytokines such as type II IFN and interleukins to further promote inflammation in tissues, facilitating viral egress and cell transformation [144,145,146].

## 4. EBV Perturbation of Programmed Cell Death

Programmed cell death is an integral part of innate antiviral defense that can be triggered by TLR, RLR, NLR, and ALR signaling. Apoptosis, necroptosis, and pyroptosis are on the expanding list of different forms of programmed cell death. Apoptosis is a cell death pathway featuring the activation of a series of initiator and executioner caspases. The initiator caspases include caspases 2, 8, 9, and 10, whereas the executioner caspases comprise caspases 3, 6, and 7 [147]. Necroptosis is another form of programmed cell death in which necrosis is mediated by the serine/threonine kinase activity of RIPK1, RIPK3, and MLKL. Morphologically distinct from apoptosis, necroptosis involves membrane rupture and the release of cellular content [148]. Pyroptosis is one form of pro-inflammatory death involving the activation of inflammasome complex and inflammatory caspases, including caspases 1, 4, 5, and 11, to mediate the formation of the pores on the host cell membrane through processing of gasdermin D [147]. Inflammatory cytokines IL-1β and IL-18 will also be matured and released from the cells to prolong the pro-inflammatory response. Cell death is the host’s last resort in the battle against viruses. In this sense, all three forms of programmed cell death serve antiviral functions to some extent. As a survival strategy, viruses have evolved countermeasures to combat different forms of cell death, especially apoptosis. As one of the most successful human viruses, EBV is well-equipped with antiapoptotic weapons to be used in different stages of its life cycle. During lytic replication, its BHRF1 protein is abundantly expressed in the early lytic phase. As the viral homolog of cellular antiapoptotic protein Bcl-2, BHRF1 binds to proapoptotic protein Bax and prevents cytochrome c release from the mitochondria, an initiation signal of apoptosis. It can also bind and inhibit a subset of proapoptotic Bcl-2 family proteins including Bid, Bim, PUMA, and Bak. The abundant expression of BHRF1 ensures the operation of the viral factory during its lytic phase of replication [149,150,151,152]. During latent infection, EBV makes use of other proteins or miRNAs to counteract apoptosis. In latency III, EBV harnesses its nuclear antigen EBNA2 to block Nur77-mediated apoptosis [153]. It also harnesses EBNA3C to inhibit p53-, IRF4/8-, and E2F1-mediated apoptosis [154,155,156]. In latency I and II, EBV miRNAs are highly expressed to modulate apoptotic pathways. Different EBV-encoded miR-BARTs have been shown to inhibit key components of apoptotic pathways, including PUMA (miR-BART5) [157]; Bim (miR-BART cluster I or II) [158]; caspase-3 (miR-BART1-3p, 16, and 22) [159,160]; Bad (miR-BART20-5p) [161]; TOMM22 (miR-BART16) [162]; FEM1B and CASZ1a (miR-BART3); OCT1 (miR-BART6); ARID2 (miR-BART8); CREBBP and SH2B3 (miR-BART16); as well as PPP3R1, PAK2, and TP53INP1 (miR-BART22) [163]. Besides miRNAs, the underexpression of LMP1 can also inhibit Fas receptor- or TRAIL receptor-induced apoptosis [164]. It can also suppress necroptosis by targeting RIPK1 and RIPK3 ubiquitination [165]. However, a high expression level of LMP1 will induce apoptosis in the host cell [166]; therefore, the LMP1 expression level is tightly controlled in the infected cells.

Although programmed cell death is generally accepted as an antiviral mechanism, recent studies have also demonstrated its proviral roles in the life cycle of gammaherpesviruses. Activated caspases 3, 6, and 8 can cleave the protein inhibitor of activated STAT1 (PIAS1) to facilitate EBV lytic reactivation. PIAS1 is a negative regulator of STAT1 signaling; it can also act as an inhibitor of the transcriptional factors involved in EBV lytic gene reactivation. Upon B cell receptor stimulation or chemical induction of lytic cycle, EBV can hijack the apoptotic caspases to cleave and inactivate PIAS1 for effective lytic replication [167]. For KSHV, a caspase-dependent mechanism is in place to block type I IFN response. The activated caspase 8 inhibits the TBK1/IKKε-IRF3 pathway to prevent the production of type I IFNs [7,168]. Taken together, apoptosis may act as a two-edged sword in EBV biology. On one hand, it can prevent the spread of the infectious materials by initiating the suicide commitment of the infected cells. On the other hand, it can help the virus escape from the cellular inhibitors to maintain effective viral infection and replication. How viruses modulate apoptosis to maintain a fine balance between its proviral and antiviral roles requires further investigations. Other than apoptosis, inflammasome activation and pyroptosis also exhibit a proviral role in EBV biology. In general, inflammasome activation by caspase 1 increases the processing of IL-1β and IL-18 and initiates the antiviral pro-inflammatory response and pyroptosis [148]. However, recent studies also suggest that activated caspase 1 can enhance the processing of the large tegument protein deubiquitinase BPLF1 of EBV [169] and can destabilize the host suppressive factor KAP1 to facilitate EBV lytic reactivation [170]. These proviral effects of caspase 1 redefine the role of inflammasome activation in EBV biology. In summary, the roles of programmed cell death in EBV biology might be more complicated than we originally expected. Further studies are required to dissect its function in the viral life cycle. How EBV fine-tunes the level of programmed cell death to its own benefit remains an important question in the next phase of analysis. The concept that caspases have proviral properties against gammaherpesviruses has emerged and received more and more support. However, the possibility that caspases could serve proviral functions independent of their roles in apoptosis and pyroptosis should also be challenged with new experiments.

**Figure 4:**
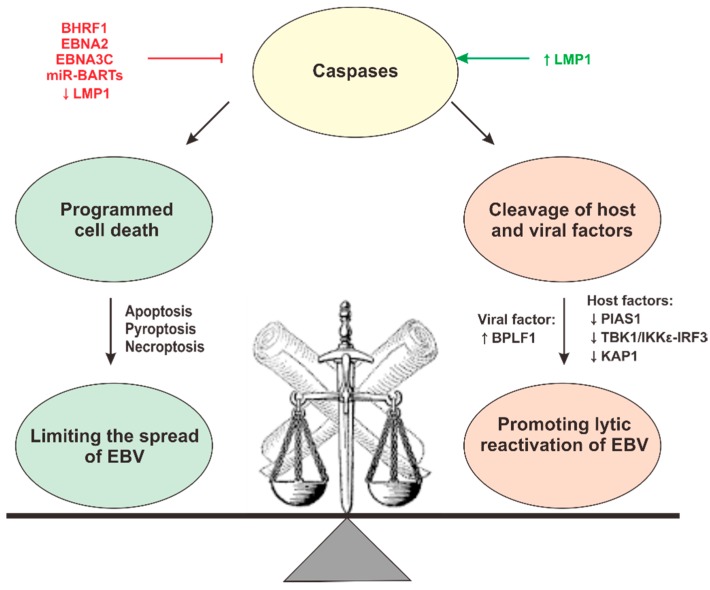
Delicate balance of proviral and antiviral roles of caspases in EBV biology. On the one hand, activation of caspases leads to programmed cell death such as apoptosis and pyroptosis, which limit the spread of virus. On the other hand, activated caspases can cleave host restriction factors such as PIAS1, TBK1/IKKε-IRF3, and KAP1 and the viral factor BPLF1 to facilitate EBV lytic reactivation. The viral inhibitors of caspase signaling are depicted in red, and the activators are depicted in green. See text for further details.

The intricate balance between the proviral and antiviral roles of caspases during EBV infection is depicted in Figure 4.

## 5. Conclusions

The interplay between EBV and innate immunity is dynamic and complex. The view that innate immunity combats EBV whereas EBV develops counterstrategies to evade innate immunity might need to be modified to reflect the dual roles of innate immunity and the multifaceted interaction between EBV and host. Whether innate immunity is a friend or foe of EBV depends on the context. Although we discuss above the interplay between EBV and innate immunity in different sections based primarily on different branches of innate immune signaling, the following trends should also be noted. First, one EBV protein can target multiple pathways in a highly orchestrated manner. For example, EBV deubiquitinase BPLF1 can suppress multiple pathways including NFκB signaling, IFN production, and IFN signaling [171]. Second, the same EBV protein can differentially target different players in the same or different pathways. The aforementioned opposite regulatory effects of LMP1 on different routes of cell signaling [90,122,166] serve as a good example of its pleiotropism. Third, innate immunity has stage-specific effects on EBV. Its roles in lytic and latent phases are different and even opposite. It will be of great interest to see whether differences could also be seen in early and late lytic phases as well as in different forms of latency states. Unfortunately, many observations documented in the literature are derived from overexpression experiments conducted in physiologically irrelevant cultured cells. Many NPC cells widely used in the research community represent contaminants or somatic hybrids with HeLa [172,173,174]. A warning alarm should be sounded loud and clear. It is very important that key experiments concerning the interaction between EBV and innate immunity be verified in physiologically relevant systems. Ideally, cells that can be naturally infected with EBV should be used and the key cellular factor should be genetically knocked out in these cells. In addition to NPC, gastric cancer is another EBV-associated epithelial cancer. The interplay between EBV and innate immunity has not been well-studied in gastric cancer cells. It will, therefore, be of interest to see how compromising innate immune signaling in these cells might affect EBV infection. Moreover, it will be of great importance to compare and contrast the perturbation of innate immune response by EBV in epithelial cells and lymphocytes. It should not be taken for granted that identical patterns will be seen in epithelial cells exposed to EBV as in infected lymphocytes. On the other hand, EBV genes that are thought to play a role in modulating innate immunity should be disrupted and the mutant viruses should be characterized in full for replication dynamics and ability to activate innate immunity. The application of new technologies including CRISPR and CRISPRa screening as well as organoids in EBV research should also help break new grounds in our understanding of the interplay between EBV and innate immunity. Some of the most important questions that should be put at the top of the priority list are as follows.

- First, are type I and type III IFNs induced and innate immunity activated in EBV-infected B cells and epithelial cells in latency I, II, and III?
- Second, can treatment with type I and type III IFNs clear EBV infection from latently infected cells?
- Third, is inflammasome activated in EBV-infected cells in latency I, II, and III?
- Fourth, can treatment with inflammasome inhibitors prevent cancer development in EBV-infected individuals?

These and other key issues in the study of EBV–host interactions will keep us busy in the coming few years.

## Figures and Tables

**Table 1:** Summary of EBV genes, their function in viral life cycle and pathogenesis, as well as their impact on innate immunity.

Gene	Function in Viral Life Cycle	Impact on Innate Immunity
LMP1	• Major viral oncoprotein • CD40 mimic	• Inhibits TLR9 transcription • Recruits and activates TRAFs • Promotes RIG-I degradation • Activates NLRP3 inflammasome • Induces IL-1α, IL-1β, and TNF-α • Induces IRF7 • Inhibits TYK2 kinase • Activates STAT3 • Inhibits apoptosis when underexpressed • Promotes apoptosis when overexpressed
LMP2A/B	B cell receptor mimic	• Increases turnover of IFNARs and IFNGRs • Activates STAT3
EBNA2	• Regulates latent gene transcription • B cell immortalization	• Prevents ISG induction • Blocks Nur77-mediated apoptosis
EBNA3C	Regulates latent gene transcription	Inhibits p53-, IRF4/8-, and E2F1-mediated apoptosis
EBER	Abundantly expressed small noncoding RNAs	• Activates RIG-I signaling and PKR • Stimulates TLR3 signaling
BZLF1 (Zta)	Lytic gene trans-activator	• Induces p65 nuclear translocation but inhibits its function • Inhibits dimerization of IRF7 • Increases turnover of IFNARs and IFNGRs • Inhibits tyrosine phosphorylation of JAK1 and JAK2
BRLF1 (Rta)	Lytic gene trans-activator	Reduces production or promotes degradation of IRF3/7
BPLF1	• Deneddylase and deubiquitinase • Tegument protein	• Inhibits TRAF6, IKKγ, and IκBα • Inhibits TRIM25-mediated RIG-I activation
BLRF2	Tegument protein	Inhibits cGAS-STING signaling
BLLF3	dUTPase	Activates TLR2 and NFκB
LF2	Rta binding protein	Inhibits dimerization of IRF7
BGLF4	Protein kinase	• Suppresses NFκB by targeting an essential coactivator UXT • Impedes formation of stable IRF3-DNA complex • Inhibits Tyr701 phosphorylation and activation of STAT1
BGLF5	Exonuclease	Shuts down TLR2 and TLR9 production
gp350	Envelope glycoprotein	Activates TLR2
BMLF1 (SM)	• mRNA export factor • ICP27 homolog	Induces STAT1 expression
BHRF1	BCL2 mimic	Inhibits apoptosis by binding to Bax
miR-BHRFs miR-BARTs	EBV-encoded miRNAs	Modulates the expression of NLRP3, MyD88, IRAK, RIG-I, STAT1, CBP, proapoptotic genes, and other targets
